# GRSF1 is an age-related regulator of senescence

**DOI:** 10.1038/s41598-019-42064-6

**Published:** 2019-04-03

**Authors:** Su-Jeong Kim, Maria Chun, Junxiang Wan, Changhan Lee, Kelvin Yen, Pinchas Cohen

**Affiliations:** Leonard Davis School of Gerontology, University of Southern California, Los Angeles, CA 90089 USA

## Abstract

Senescent cells that accumulate in multiple tissues with age are thought to increase pathological phenotypes. The removal of senescent cells can improve lifespan and/or healthspan in mouse models. Global hypomethylation and local hypermethylation in DNA are hallmarks of aging but it is unclear if such age-dependent methylation changes affect specific genes that regulate cellular senescence. Because mitochondria play important roles in aging and senescence, we tested if age-associated methylation changes in nuclear-encoded mitochondrial proteins were involved in regulating cellular senescence. Here, we examined the role of hypermethylation of the G-rich sequence factor 1 (GRSF1) promoter region, a mitochondrial RNA binding protein, in replication- and doxorubicin-induced cellular senescence. GRSF1 expression was lower in senescent fibroblasts, and GRSF1 knockdown induced senescence in human primary fibroblasts. These results suggest that the age-dependent hypermethylation of GRSF1 reduces its expression, which can potentially contribute to cellular senescence during aging.

## Introduction

Cellular senescence not only plays an important role in wound healing and cancer suppression, but also contributes to age-related tissue dysfunction^1^. Senescent cells accumulate with age in multiple tissues including skin, liver, fat, kidney, and the cardiovascular system in various species including human, mouse, and numerous multicellular organisms^2,3^. Senescent cells show irreversible cell cycle arrest and secrete various factors of the senescence-associated secretory phenotype (SASP), including proinflammatory cytokines, chemokines, proteases, and growth factors^4^. These SASP factors maintain cell cycle arrest in an autocrine manner and induce senescence in neighboring cells in a paracrine manner^5^. Telomere shortening, persistent DNA damage response, and mitochondrial dysfunction are known to drive senescence *in vitro* and *in vivo*^6,7^. In fact, mitochondrial dysfunction can induce senescence with a distinct SASP, which is referred to as mitochondrial dysfunction-associated senescence (MiDAS)^8^. Cells that exhibited MiDAS had a reduced NAD+/NADH ratio triggered by AMPK-dependent p53 activation that led to a unique SASP that lacked IL-1^8^. Nonetheless, MiDAS shares major characteristics with other types of senescence such as cell cycle arrest and senescence-associated β-galactosidase (SA-β-gal) expression^8^.

Senescent cells are metabolically active in order to support the energy demanding processes including SASP production and autophagy induction. Certain senescent cells, including replicatively senescent cells, depend more on glycolysis, whereas others, including therapy- and oncogene-induced senescent cells, rely more on mitochondrial respiration^9,10^. Our group recently compared the mitochondrial characteristics of doxorubicin-induced senescent human fibroblasts to replicatively senescent cells^11^. We found that doxorubicin-induced senescent cells have increased mitochondrial number and respiration. They also show differential regulation of mitochondrial DNA methylation, mitochondrial-encoded protein levels, and mitochondrial-derived peptides (MDPs). The mitochondrial complex I proteins and MDPs, humanin and MOTS-c, were elevated in doxorubicin-induced senescent cells. While examining nuclear-encoded mitochondrial gene expression in senescent fibroblasts, we consistently found reduced expression of G-rich sequence factor 1 (GRSF1), a mitochondrial RNA binding protein, in senescent cells. Here, we report the biological significance of GRSF1 during senescence.

Basic biological processes associated with aging and age-associated phenotypes include cellular senescence, genomic instability, mitochondrial dysfunction, and epigenetic alteration^12^. DNA methylation is a well-studied epigenetic modification that occurs mostly on the 5-carbon of cytosine (5-methylcytosine; 5mC) residues in CpG dinucleotides^13^. Mammalian cells undergo global DNA hypomethylation and local DNA hypermethylation on 5mC with age^14^. Such hypomethylation occurs mainly in repetitive regions of the genome that correlate with constitutive heterochromatin, whereas local DNA hypermethylation occurs primarily at promoter CpG islands. Recent studies demonstrated that specific sets of CpGs can predict the biological age with considerable accuracy (called the epigenetic clock)^15–18^. These studies suggest that the DNA methylation pattern can serve as a molecular marker of aging, which can be used to predict, monitor, and provide insight into age-associated diseases and interventions. The pattern of DNA methylation and local hypermethylation may inactivate specific transcriptional programs^19,20^. However, it is unclear which specific genes are directly affected by DNA methylation alterations during aging. In addition to methylation-based epigenetic alterations, mitochondria are also potential drivers of aging phenotypes^12,21^. A decline in mitochondrial quantity and function is associated with aspects of aging, including cellular senescence, inflammation, and age-related degeneration^8,22–24^. As DNA methylation and mitochondrial dysfunction are known to contribute to aging, we hypothesized that age-related hypermethylation at promoter CpG islands may occur in nuclear genes that encode mitochondrial proteins and potentially inhibit their expression, thereby causing mitochondrial dysfunction. As mentioned above, doxorubicin-induced senescent cells downregulated GRSF1, a mitochondrial RNA binding protein. Because the GRSF1 promoter has CpG islands, we hypothesized that it may be a potential target of age-related hypermethylation.

GRSF1 was originally cloned as a cytosolic RNA binding protein that binds to RNAs with G-rich elements^25^. GRSF1 contains three RNA-recognition motifs and regulates the translation of mRNA species of both cellular and viral origin^25^. GRSF1 binds to the 5′-untranslated region (5′ UTR) of the influenza virus nucleocapsid mRNA and increases its translation^26^. It also binds to the 5′ UTR of the mitochondrial hydroperoxide glutathione peroxidase 4 (GPX4) mRNA and upregulates its translation during embryonic brain development^27^. Moreover, GRSF1 can also act as an RNA chaperone (*e*.*g*. *myc*)^28^. GRSF1 localizes to mitochondria^29^ and proteomic analysis identified GRSF1 as a partner of C12orf65, a protein involved in mitochondrial translation^30^. The GRSF1 gene encodes two splice variants, and both isoform 1 and isoform 2 contain three RNA-binding motifs. Isoform 1 contains a mitochondrial localization signal (MLS) and localizes to mitochondria, whereas isoform 2 does not and remains in the cytosol^29^. Isoform 1 participates in mitochondrial ribosome assembly and regulates RNA processing, particularly t-RNA-less RNA precursors, in the mitochondrial RNA granules^29,30^. GRSF1 binds to RNAs derived from the light strand of mtDNA, which encodes ND6 and two long noncoding RNAs (ncND5 and ncCYTB)^30^. It is also involved in the trafficking of a nuclear-encoded long noncoding RNA, RMRP (RNA Component Of Mitochondrial RNA Processing Endoribonuclease) into mitochondria^31^. RMRP is important for mtDNA replication and RNA processing^32,33^. Thus GRSF1 knockdown prevents the mitochondrial translocation of RMRP, thereby, leading to the reduction of mtDNA replication and mitochondrial respiration^31^.

Here, we show that the GRSF1 promoter becomes increasingly methylated in human senescent fibroblasts. Such methylation inhibited GRSF1 expression and induced cellular senescence.

## Results

### Methylation of the GRSF1 promoter is increased during cellular senescence

**Figure 1:**
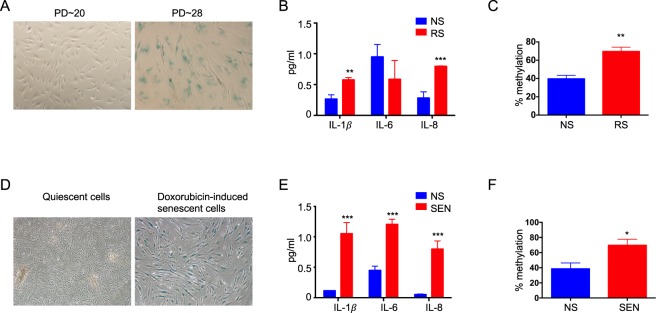
Methylation of the GRSF1 promoter increases during cellular senescence. (**A**–**C**) Quiescent (population doubling (PD) < 20) and replicatively-senescent (PD > 28) HDFa were assayed for (**A**) SA-β-GAL activity, (**B**) SASP: IL-1β, IL-6, and IL-8 secretion and (**C**) DNA methylation levels at the GRSF1 promoter. (**D**–**F**) Quiescent and doxorubicin-induced senescent HDFa were assayed for (**D**) SA-β-GAL activity, (**E**) SASP: IL-1β, IL-6, and IL-8 secretion, and (**F**) DNA methylation levels at the GRSF1 promoter. Data are reported as mean ± SEM of three to four independent experiments. Statistical significance was determined by Student’s *t*-tests. *p < 0.05, **p < 0.01, ***p < 0.001. Abbreviations: NS for Non-Senescent cells (quiescent); RS for replicative-senescent cells; SEN for senescent cells.

We tested our hypothesis that age-related DNA hypermethylation occurs at the promoter of GRSF1 using replicative and doxorubicin-induced cellular senescence models. Primary human dermal fibroblasts (HDFa) were cultured until proliferation ceased and replicative senescence was visually examined by SA-β-gal staining (Fig. 1A). Most HDFa with higher population doubling number (PD) (>28) showed positive SA-β-gal staining, whereas those with lower PD (<20) did not (Fig. 1A). We further compared the levels of a small subset of SASP factors in the conditioned media from non-senescent (quiescent) and replicatively senescent cells. IL-1β and IL-8 levels were higher in senescent cells (Fig. 1B). Interestingly, GRSF1 promoter methylation was increased in senescent cells by two-fold (Fig. 1C). We further tested if GRSF1 promoter methylation also increased in stress-induced premature senescent cells, using doxorubicin-treated HDFa. After 14 days, most HDFa stained positive for SA-β-gal expression, whereas the non-senescent (quiescent) did not (Fig. 1D), and IL-1β, IL-6, and IL-8 levels were elevated in the conditioned media of senescent cells (Fig. 1E). Notably, the GRSF1 promoter methylation was also increased in doxorubicin-induced senescent cells by two-fold (Fig. 1F).

### GRSF1 expression is decreased in senescent cells

**Figure 2:**
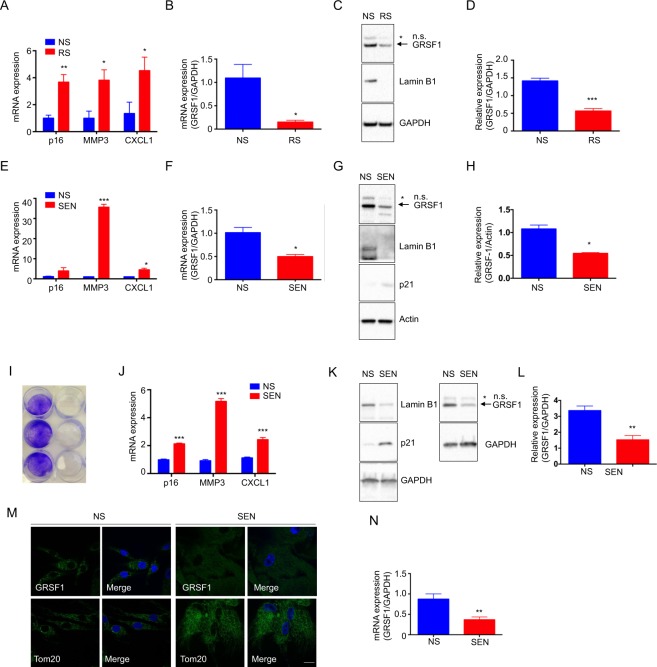
GRSF1 expression decreases in cellular senescence. (**A**–**D**) Quiescent and replicative-senescent HDFa: (**A**) mRNA expression of p16, MMP3, and CXCL1 by qRT-PCR, (**B**) mRNA levels of GRSF1 by qRT-PCR, (**C**) GRSF1 levels (representative western blots); GAPDH was used as a loading control and Lamin B1 is used as a senescent marker. Full-length blots are presented in Supplementary Fig. 3A. (**D**) Quantification of GRSF1 in western blots. (**E**–**H**) Quiescent and hydrogen peroxide-induced senescent HDFa: (**E**) mRNA expression of p16, MMP3, and CXCL1 by qRT-PCR, (**F**) mRNA levels of GRSF1 by qRT-PCR, (**G**) GRSF1 levels (representative western blots); GAPDH was used as a loading control and Lamin B1 and p21 is used as a senescent marker. Full-length blots are presented in Supplementary Fig. 3B. (**H**) Quantification of GRSF1 in western blots. (**I**–**N**) Non-senescent and doxorubicin-induced senescent HDFa: (**I**) Clonogenic assay for proliferation was performed. (**J**) mRNA levels of p16, MMP3, and CXCL1 were determined by qRT-PCR. (**K**) Representative western blots of GRSF1. GAPDH is used as a loading control and Lamin B1 and p21 are used as senescent markers. Full-length blots are presented in Supplementary Fig. 3C. (**L**) Quantification of GRSF1 in western blots. (**M**) Representative images of GRSF1 (green), Tom20 (green), and Hoechst 33258 (blue; nucleus) immunostaining in quiescent and doxorubicin-induced senescent cells. Scale bar, 20 µm. Other supporting fields of cells were shown in Supplementary Fig. 4. (**N**) mRNA levels of GRSF1 were determined by qRT-PCR. Data are reported as mean ± SEM of three to four independent experiments. Significant differences were determined by Student’s t-tests. *p < 0.05, **p < 0.01, and ***p < 0.001. Abbreviations: NS for Non-senescent cells (quiescent); RS for replicative-senescent cells; SEN for senescent cells.

Typically, DNA methylation at the promoter region is accompanied by an epigenetically mediated silencing of gene transcription that suppresses gene expression^34^. We therefore measured the levels of GRSF1 mRNA and protein in replicatively senescent HDFa (Fig. 2B–D). One of the well-established characteristics of both replicatively and stress-induced premature senescence (*e*.*g*. DNA damage) is the activation of the p53-p21 and/or p16-Rb pathway. Indeed, replicatively senescent cells expressed p16 four times higher than non-senescent cells (Fig. 2A). Production of SASP in replicative senescence is indicated by increased levels of metalloproteinase-3 (MMP3) and C-X-C Motif Chemokine Ligand 1 (CXCL1) (Fig. 2A). GRSF1 mRNA and protein levels were substantially reduced in replicatively senescent HDFa compared to non-senescent quiescent cells (Fig. 2B–D). To test if these observations were senescence model-specific, we extended the measurements to stress-induced premature senescence induced by hydrogen peroxide or doxorubicin in HDFa. Hydrogen peroxide-induced senescence showed little p16 expression (Fig. 2E), but increased p21 expression (Fig. 1G), suggesting a higher reliance on the p53-p21 pathway rather than the p16-Rb pathway. A subset of SASP factors, MMP3 and CXCL1, were highly expressed in hydrogen peroxide-induced senescent cells compared to quiescent cells (Fig. 2E), and the mRNA and protein levels of GRSF1 were reduced by 50% in senescent cells (Fig. 2F–H). Next, doxorubicin-induced senescent cells, which rarely form clonogenic colonies, (Fig. 2I), exhibited increased expression of p16, MMP3, and CXCL1 (Fig. 2J), but reduced protein levels of GRSF1 (Fig. 2K,L). In addition, we visually confirmed that mitochondrial GRSF1 levels significantly decreased in senescent cells using immunofluorescence imaging (Fig. 2M). Along the same lines, GRSF1 mRNA levels were also decreased in doxorubicin-induced senescent HDFa (Fig. 2N). We conducted a systematic analysis of available RNA sequencing databases using the Illumina BaseSpace Correlation Engine to identify other cell lines and stimuli that change GRSF1 expression during senescence (Supplemental Table 1). Ras-oncogene-induced senescent IMR-90 (lung fibroblast) cells showed reduced GRSF1 mRNA expression by 2-fold (Supplemental Table 1). The mRNA levels of GRSF1 in were lower in human skin fibroblasts with higher PD by 1.8-fold (Supplemental Table 1). Adriamycin-induced senescent human skin fibroblasts and doxorubicin-induced senescent liposarcoma cells also showed lower GRSF1 mRNA levels by 1.5 and 1.3-fold, respectively (Supplemental Table 1). Therefore, GRSF1 expression is negatively regulated by DNA methylation in various cellular senescent models.

### GRSF1 levels decline during aging

**Figure 3:**
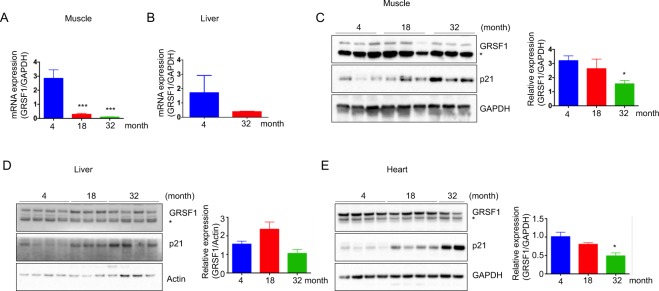
GRSF1 levels decline in mice tissues during aging. mRNA levels of GRSF1 were determined by qRT-PCR in mouse (**A**) skeletal muscle and (**B**) liver. Quantification and representative western blots of GRSF1 in mouse (**C**) skeletal muscle, (**D**) liver and (**E**) heart at 4, 18, and 32 months. p21 was used as a senescence marker. Additional western blots of GRSF1 in mouse skeletal muscle are presented in Supplementary Fig. 2. ^*^Indicates GRSF1 isoform 2 localized in the cytosol. Statistical significance determined by one-way ANOVA followed by Tukey’s post hoc test. *p < 0.05, ***p < 0.001.

Senescent cells accumulate with age and are implicated in age-related pathologies. Elimination of senescent cells can delay age-related diseases, including hepatic steatosis, cataract formation, skin disorder, and age-related fat loss in mouse models^35–39^. We measured GRSF1 in young and aged mouse tissues. The level of p21, which is involved in the induction of senescence and serves as a senescence marker, were gradually increased with age at 4, 18, and 32 months in skeletal muscle, liver, and heart. Skeletal muscle showed a significant reduction of GRSF1 mRNA expression at 32 months compared to 18 months, whereas liver showed similar levels at 4 and 32 months (Fig. 3A,B). Age-dependent accumulation of senescent cells vary depending on the tissue type^40^. Although the senescent cells accumulation is relatively small in skeletal muscle and heart, the protein levels of GRSF1 in the skeletal muscle and heart were reduced at 32 months compared to 4 months, whereas it was stable in the liver (Fig. 3C–E). The increase of p21 expression during aging is less in the liver compared to the skeletal muscle and heart (Fig. 3D) GRSF1 levels are inversely correlated with p21 expression. The gap between the large decline in mRNA and the small decline in protein levels remains unclear. It is also uncertain if GRSF1 levels only decline in senescent and not in non-senescent, aged cells. Further studies analyzing single-cell RNA sequencing analysis in old mice tissues would advance our understanding on GRSF1 levels during aging.

### GRSF1 knockdown induces senescence in primary fibroblasts

**Figure 4:**
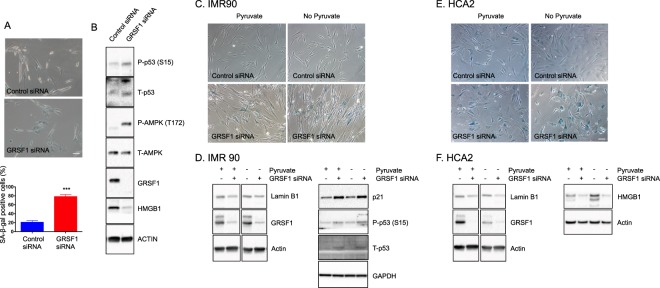
Knockdown of GRSF1 induces senescence in primary fibroblasts. (**A**) Quantification and representative images of SA-β-gal positive cells in control siRNA and GRSF1 siRNA-treated WI38 cells. A total of 100–130 cells were counted. Other supporting fields of cells were shown in Supplementary Fig. 5A. (**B**) Western blots of senescence-related proteins in control siRNA and GRSF1 siRNA-treated WI38 cells. Full-length blots are presented in Supplementary Fig. 6. (**C**) Representative images of SA-β-gal positive cells in control siRNA and GRSF1 siRNA-treated IMR90 cells. (**D**) Western blots of Lamin B1, p21, and p53, senescent markers in control siRNA and GRSF1 siRNA-treated IMR90 cells. (**E**) Representative images of SA-β-gal positive cells in control siRNA and GRSF1 siRNA-treated HCA2 fibroblasts. (**F**) western blots of Lamin B1, p21, HMGB1, senescent markers in control siRNA and GRSF1 siRNA-treated HCA2 fibroblasts. (N = 3–5) Other supporting fields of cells were shown in Supplementary Fig. 5B,C. Full-length blots are presented in Supplementary Fig. 7A,B.

So far, we showed that GRSF1 levels were decreased during senescence and aging in a tissue-specific manner. To understand the causal relationship between GRSF1 reduction and senescence, we tested if GRSF1 (isoform 1) knockdown using siRNA can induce cellular senescence in primary lung fibroblasts (WI-38). GRSF1 knockdown caused an increase in SA-β-gal positive WI-38 cells (Fig. 4A) and increased phosphorylated p53 levels, which is a senescence marker (Fig. 4B). HMGB1 (high mobility group box 1) modulates nuclear gene expression, but it is released to the extracellular milieu in senescent human and mouse cells in culture and *in vivo* in a p53 dependent manner^41^. Cellular HMGB1 depletion due to its secretion is a senescence marker. The level of HMGB1 was decreased in GRSF1 knockdown cells compared to control cells (Fig. 4B). Furthermore, GRSF1 knockdown caused a statistically significant increase in IL-6 production (0.75 pg/ml ± 0.0017, n = 3) compared to control cells (0.57 pg/ml ± 0.03, n = 3) and a negative trend in IL-8 production (5.35 ± 1.29 pg/ml, n = 3) compared to control cells (7.68 ± 0.349 pg/ml, n = 3). To test if these results were specific to WI-38 cells, we also tested HCA2 (human foreskin fibroblasts) and IMR90 (human fetal lung fibroblasts). GRSF1 knockdown increased the number of SA-β-gal positive HCA2 and IMR90 cells, decreased lamin B1 expression, increased p21 and p-p53 levels, and decreased HMGB1 (Fig. 4D–F), which are all senescence markers. Together, these results suggest that the GRSF1 plays a role in regulating the induction of cellular senescence. MiDAS has a distinct SASP phenotype driven by the AMPK-p53 pathway^8^. GRSF1 modulates posttranscriptional regulation of mitochondrial-encoded gene expression, and GRSF1 knockdown leads to mitochondrial RNA instability, abnormal ribosome assembly, and decreased mitochondrial respiration^31^. Therefore, GRSF1 knockdown in fibroblasts can result in mitochondrial dysfunction leading to mitochondrial dysfunction-associated senescence. We also examined AMPK activation along with p53 activation in GRSF1 siRNA-treated cells and found that AMPK phosphorylation is elevated in GRSF1 siRNA-treated cells (Fig. 4B). As pyruvate can inhibit MiDAS, the senescence phenotype were increased in the presence or absence of pyruvate (Fig. 4D,F). Taken together, GRSF1 knockdown plays a role in inducing senescence in fibroblasts.

## Discussion

In this study, we examined whether DNA methylation at the promoter of GRSF1, a mitochondrial RNA binding protein, increased during cellular senescence. The expression level of GRSF1 isoform 1, which localizes to mitochondria, decline in senescent cells and aged mouse skeletal muscle and heart, but not in the liver. GRSF1 knockdown is sufficient to induce senescence in human fibroblasts. GRSF1 plays crucial roles in mitochondrial replication, translation, and respiration^29–31^. GRSF1 knockdown was shown to increase mitochondrial RNA instability, lessen mitochondrial translation, and lessen respiration^31^. Thus, GRSF1 knockdown-induced senescence may be caused by mitochondrial dysfunction. This study suggests that GRSF1 is a potential target of hypermethylation observed in aging and can be important for mitochondrial alteration during aging.

DNA methylation is a well-established epigenetic modification due to relatively simple sample preparation and availability of cost-effective techniques, including bisulfite conversion coupled to PCR, pyrosequencing, microarrays, and deep sequencing^42^. Most epigenetic clock studies use bisulfite conversion coupled to microarray-type beadchip analysis, but they are still missing various chromosomal regions, including GRSF1, despite of the large coverage of CpG sites. Here, we quantified DNA methylation levels at the promoter region of selected genes that are (i) important for mitochondrial function and are (ii) not present in the widely-used microarray-type beadchips. We used Methylation Sensitive Restriction Enzymes (MSREs), rather than bisulfite conversion, to distinguish methylated from non-methylated DNA^43^. The methylation levels are determined by qPCR performed using specific primers flanking the region of methylation targets^43^. It is a rapid, simple, and reliable tool to screen single- and multi-locus DNA methylation, although the selective sites are limited because the region must include sequences that are recognized by MSREs. The primers we designed for the GRSF1 promoter region cover three specific MSREs sites, that include a total of 27 CpG sites. To reveal the exact methylation pattern in all 27 CpG sites of this promoter region, we need further analysis, such as bisulfide conversion followed by pyrosequencing or deep sequencing. Further studies examining the exact methylation patterns in GRSF1 promoter during senescence and aging will give us insight into a new epigenetic clock to understand the biology of aging.

A decline of mitochondria activity is associated with aging^44^. We tested if reduced GRSF1 expression resulted in a general reduction of mitochondrial protein levels along with a decline in mitochondrial activity. There was no change in the levels of the mitochondrial outer membrane protein (Tom20) or the matrix protein (HSP60) in replicatively, hydrogen peroxide-induced, and doxorubicin-induced senescent cells (Supplemental Fig. 1A–C). Previously, we reported increased level of MDPs and a protein consisting of mitochondrial respiratory chain complex I^11^. This suggests that GRSF1 expression can be modulated by biological processes during senescence and aging. Examining whether overexpression of GRSF1 can delay cellular senescence and if any interventions can increase the levels of GRSF1 during aging would be important in translating these findings into a therapy for delaying the senescence phenotype.

GRSF1 plays multiples roles in the mitochondria: mitochondrial ribosome biosynthesis, mitochondrial translation, and mitochondrial noncoding RNA binding. GRSF1 knockdown-induced senescence, therefore, could be underlined by mitochondrial translational defects or the loss of mitochondrial noncoding RNA. In fact, cellular models of mitochondrial disruption (*e*.*g*. depleting mtDNA by culture in ethidium bromide (rho0 cells), treating with electron transport chain inhibitors, rotenone or antimycin A, and knocking down mitochondrial SIRT3 and 5) were shown to induce senescence^8^. Because GRSF1 knockdown can result in translation inhibition, we examined whether other mitochondrial translation machinery were also downregulated during senescence. However, the expression of TUFM, mitochondrial Tu translation elongation factor, remained the same in senescent fibroblasts (Supplemental Fig. 1A–C), indicating other possible ways of inducing senescence by GRSF1 knockdown. GRSF1 is a RNA binding protein that primarily binds to the light strand of mitochondrial DNA, which encodes ND6 and two mitochondrial long noncoding RNA, ncND5 and ncCYTB^30^. The long noncoding RNAs were known as RNAs with no protein-coding capacity. Emerging studies show they are key regulatory molecules in the cells. The long noncoding RNAs play crucial roles such as modulating RNA processing, transcription, and gene editing, and they are processed to yield small RNAs and peptides^45^. However, the roles of mitochondrial long noncoding RNAs are still unknown^46^. GRSF1 also regulates the trafficking of nuclear-encoded long noncoding RNAs into the mitochondria^31^. Senescent cells showed lower GRSF1 expression, thus the levels of mitochondrial long noncoding RNAs, ncND5, and ncCYB and nuclear-encoded long noncoding RNAs may be lower in senescent cells. Further studies to determine whether mitochondria-localized long noncoding RNAs are involved in senescence processes will give us insights on the key molecules involved in cellular senescence.

In summary, our studies suggest that GRSF1 has a key role in cellular senescence and may be involved in aging. Consequently, GRSF1 reduction induced senescence in several human fibroblasts. These results suggest that the mRNA binding protein GRSF1 and its downstream molecules can be key molecules that regulate senescence.

## Methods

### Reagents and Antibodies

Doxorubicin, hydrogen peroxide(Sigma, St. Louis, MO, USA) were used for inducing senescence status *in vitro*. siRNA targeting GRSF1 isoform 1 and control siRNA were designed and ordered from Dharmacon (Lafayette, CO, USA). The following antibodies were used in this study: anti-GAPDH antibody (Cat. #5174S), anti-phospho-p53 (Ser15) antibody (Cat. #9284), anti-phospho-AMPKα (Thr172) antibody (Cat. #2535), anti-Lamin B1 antibody (Cat. #12586), anti-rabbit IgG, HRP-linked antibody (Cat. #7074), and anti-mouse IgG, HRP-linked antibody (Cat. #7076). These antibodies are manufactured by Cell Signaling Technology (Danvers, MA, USA). Anti-GRSF1 (Cat. #HPA036985; Sigma), anti-β-actin antibody (Cat. A5316; Sigma), anti-p53 antibody (Cat. # sc-126; Santa Cruz Biotechnology, Dallas, Texas, USA), anti-Tom20 antibody (Cat. #SC-17764; Santa Cruz Biotechnology), anti-HMGB1 antibody (Cat. # ab18256; Abcam, Cambridge, MA, USA), anti-p21 (Cat. # 556430; BD Biosciences, San Jose, CA), anti-TUFM (Cat. # ab67991; Abcam), and anti-hsp60 (Cat. # sc-1052; Santa Cruz Biotechnology), were also used.

### Cell culture and treatment

Primary dermal fibroblast: Normal human adult cells (HDFa) were purchased from ATCC (Cat#. PCS-201-012; Manassas, VA, USA) and were cultured in Fibroblast Basal Medium supplemented with Fibroblast Growth Kit-Low serum (ATCC) at 37 °C in 5% CO_2_. IMR90 and HCA2 cells, human primary fibroblasts from lung and neonatal foreskin, were gifts from Dr. Judith Campisi. The IMR90 and HCA2 primary cells were cultured in high glucose Dulbecco’s modified Eagle’s medium (DMEM; Life Technologies, Waltham, MA, USA) supplemented with 10% fetal bovine serum (FBS; Omega Scientific, Tarzana, CA, USA) at 37 °C in 5% CO_2_. For replicative senescence, primary cells were passaged every 3 days until they reached 20–25 population doublings (PDs) and used for the proposed experiments compared to cells at 5–6 PDs. For stress-induced premature senescence, 250 nM doxorubicin-and  200 μM hydrogen peroxide, -induced senescence were used to induce senescence^47,48^. Briefly, primary cells were treated with 250 nM doxorubicin for 24 h, and the medium was then replaced with complete medium every 3 days for 10–14 days. Primary cells were treated with 200 μM hydrogen peroxide for 2 hr and then replaced with complete medium, and the treatment was repeated with hydrogen peroxide every 3 days until 7–10 days. For siRNA transfection, cells were seeded on 6-well plates, and were transfected with siRNA using Lipofectamine® RNAiMAX according to the manufacturer’s instruction (ThermoFisher Scientific, Wilmington, DE, USA). To get a knockdown during the senescence development, we repeated siRNA transfection every 3 to 4 days for 10–12 days.

### Western blot analysis

Cells were lysed with RIPA Lysis and Extraction Buffer (ThermoFisher Scientific) plus the Halt protease & phosphatase inhibitor cocktail (ThermoFisher Scientific). The lysates were incubated on ice for 10 min then homogenized using a sonicator, and the supernatant was collected by centrifugation at 15,000 × g for 15 min at 4 °C. For tissue, dissected skeletal muscles were lysed with RIPA buffer and homogenized using a tissue homogenizer followed by sonication. The supernatant was collected by centrifugation at 15,000 × g for 15 min at 4 °C. Protein content in the cellular lysates was quantified using the Pierce™ BCA Protein Assay Kit (ThermoFisher Scientific). Predetermined amounts of proteins (10–30 μg) were separated on 8–16% SDS-PAGE gels and blotted onto PVDF membranes (Biorad, Hercules, CA, USA). Membranes were incubated with primary antibody at 4 °C overnight according to the manufacturer’s instructions. After several washes with Tris-buffered saline containing 0.1% Tween-20, membranes were incubated at room temperature for 1 hr with the appropriate HRP-conjugated secondary antibody. Clarity™ Western ECL substrate (Biorad) was used for detecting specific bands. Membranes were imaged on a Bio-Rad ChemiDoc XRS^+^ imager. If necessary, relative intensities of the bands in each condition were measured using Image J, a free software program provided by National Institute of Health (Bethesda, Maryland, USA).

### Immunocytochemistry

Primary dermal fibroblast cells on coverslips were fixed with 4% paraformaldehyde for 10 min at room temperature. After fixation, the cells were permeabilized with 0.2% Triton X-100 in phosphate-buffered saline (PBS) for 10 minutes at room temperature and were blocked in PBS containing 0.2% Triton X-100 and 1% bovine serum albumin (BSA) for 1 hour at room temperature. Cells were then incubated with rabbit anti-GRSF1 antibody (1:100; Sigma) or mouse anti-Tom20 antibody (1:100; Santa Cruz Biotechnology) in PBS containing 0.2% Triton X-100 and 1% BSA at 4 °C overnight. After three washes with PBS, the cells were further incubated with Alexa Fluor 488-conjugated donkey anti-rabbit IgG (1:200; Invitrogen) or Alexa Fluor 488-conjugated donkey anti-mouse IgG (1:200; Invitrogen) in PBS containing 0.2% Triton X-100 and 1% BSA for 1 hour at room temperature in dark. Nuclei were stained for 5 minutes at room temperature in PBS containing Hoechst 33258 (2 mg/ml; Invitrogen). Coverslips were mounted with ProLong Gold antifade reagent (Invitrogen) and observed under an LSM780 confocal microscope (Carl Zeiss, Germany).

### DNA extraction and measurement of mtDNA methylation

Genomic DNA was extracted with DNeasy Blood & Tissue Kits (Qiagen, Valencia, CA, USA). 20 ng of genomic DNA were used for mtDNA methylation. mtDNA methylation was measured by OneStep qMethyl Kit (ZYMO RESEARCH), which contain Methylation Sensitive Restriction Enzymes (MSREs: AccII, HpaII, and HpyCH4IV) according to the manufacturer’s instruction. Briefly, each 20 ng DNA is divided into two tubes: a test reaction and a reference reaction. In test reaction, DNA was digested with MSREs while in the reference reaction was not. The DNA from both samples is then amplified using real-time PCR machine (Biorad), then C_T_ values for test and reference DNA samples were quantified for the percent methylation levels. Percent methylation is determined by the equation: 100 × 2^−ΔCT^. ΔC_T_ is the average C_T_ from the test reaction minus the average C_T_ from the reference reaction. The primers used for amplification are: GRSF-1 promoter Forward primer (5′GACAGGCTCCTTCCCTACAA3′), and Reverse primer (5′ACCTCGCATCTTCTTGCTTT3′).

### RNA extraction and qRT-PCR

Total RNA was extracted from cells and tissues using Direct-zol RNA MiniPrep Plus (ZYMO RESEARCH, Irvine, CA, USA). 500 ug-1 mg of RNA was used for reverse transcription by using SuperScript IV Reverse Transcriptase and oligo dT_20_ primers (ThermoFisher Scientific) according to the manufacturer’s instruction. Ssoadvanced Universal SYBR green supermix was used to amplify cDNA and quantify the relative gene expression analysis. The 2^−ΔΔCT^ method is used for relative gene expression analysis. This method shows the fold increase (or decrease) of the target gene in the test sample relative to the calibrator sample and is normalized to the expression of a reference gene. First, we normalized the C_T_ of the target gene to that of the reference gene for both the test and calibrator sample (ΔC_T_). Secondly, we normalized the ΔC_T_ of the test sample to the ΔC_T_ of the calibrator sample (ΔΔC_T_). Finally, we calculated the expression ratio, 2^−ΔΔCT^. The primers used for amplification are: GRSF-1 isoform 1 Forward primer (5′GGCTGCGGCTGTAACTG 3′), and Reverse primer (5′CCGACGGGATAGAGCCA 3′); ACTIN Forward primer (5′CCAACCGCGAGAAGATGA3′), and Reverse primer (5′TCCATCACGATG CCAGTG3′); GAPDH Forward primer (5′GGTGTGAACCATGA GAAGT AT GA3′), and Reverse primer (5′GAGTCCTTCCACGATACCAAAG3′); RPS13 Forward primer (5′CATGGCTCGCTC GGTGAC3′), and Reverse primer (5′CAGTTCAGTATGTTCGGCTTCC3′); P16 Forward primer (5′GTGGACCTGGCTGAGGAG3′), and Reverse primer (5′CTTTCAATCGGGGATGTCTG3′); MMP3 Forward primer (5′CAAAACATATTTCTTTGTAGAGGACAA3′), and Reverse primer (5′TTCAGCTATTTGCTTGGGAAA3′); CXCL1 Forward primer (5′GCTGAACAGTGACAAATC CAAC3′), and Reverse primer (5′CTTCAGGAACAGCCACCAGT3′).

### SA -β-GAL assay

SA-β-Gal activity was measured by a Senescence Detection Kit (BioVision, Milpitas, CA, USA), according to the manufacturer’s instruction. Briefly, cells were fixed for 10 mins at room temperature. Cells were stained with a solution containing X-gal at 37 °C overnight. The next day, cells were visualized using Olympus BX40 equipped with an OLY-105 camera (200X magnification).

### Clonogenic assay

0.05% crystal violet in 25% methanol was used for staining cells. Cells were fixed for 5 min with 100% methanol and stained with crystal violet solution for 5 min. After staining, cells were washed by soaking gently in a 2 L beaker filled with distilled water. Water was changed and washed twice. Excessive water was removed and allowed to dry. Plates were imaged using a scanner.

### SASP measurements

IL-6, IL-1β, IL-8, and TNFα levels in the conditioned medium were determined using the V-PLEX pro-inflammatory panel 1 human custom cytokine (MESO SCALE DISCOVERY, Rockville, MD, USA) according to the manufacturer’s protocols. Measurements were normalized to the number of cells in each sample.

### Animals

Frozen muscle, liver, and heart tissues from female C57BL/6 mice at 4, 18, and 32 months of age were obtained from the National Institute on Aging Aged Rodent Tissue Bank. The tissues were shipped to Dr. Cohen at USC.

### Statistical analysis

Data are presented as mean ± S.E.M. Significant differences were determined by Student’s *t*-tests, one-way ANOVA followed by Tukey’s *post hoc* test using GraphPad Prism 5 software. Values of *<0.05, **<0.01, ***<0.001 were considered statistically significant.

## Supplementary information

Supplementary table and figures
