# Supplementary material for: GRSF1 is an age-related regulator of senescence
Source: Sci Rep. 2019 Apr 3;9:5546. doi: 10.1038/s41598-019-42064-6 (PMC6447602; doi:10.1038/s41598-019-42064-6)

GRSF1 is an age-related regulator of senescence

Su-Jeong Kim, Maria Chun, Junxiang Wan, Changhan Lee, Kelvin Yen, Pinchas Cohen*

Leonard Davis School of Gerontology, University of Southern California, Los Angeles, CA 90089, USA

Supplemental table 1. Search results of GRSF1 expression levels in the available database using Illumine BaseSpace Correlation Engine

1st Author	Journal	Samples	Type of Senescence	Fold change	P-value	Test expression	Control expression	Q-value	method
Shin-ichiro Takebayashi	Aging Cell (2015), Vol.14	IMR90-Ras (n=2/group) vs IMR90	Oncogene induced senescence	-2.11	0.0484	1161.9	3393.5	none	Affymetrix U133 Plus 2.0 microarray
Maggie Purcell	Cell cycle (2014), Vol.13	Skin Fibroblasts from a patient with Li-Fraumeni Syndrome at passages 17-19 vs 10-12 (n=4/group)	Replicatively induced senescence	-1.884	6.4E-5	11.9302	26.4147	0.0016	Illumina HiSeq 2000, RNA-seq
Maggie Purcell	Cell cycle (2014), Vol.13	Skin Fibroblasts from a patient with Li-Fraumeni Syndrome adriamycin-induced senescence vs control (n=4/group)	Therapy induced senescence	-1.5229	0.0016	13.9997	23.008	0.0071	Illumina HiSeq 2000, RNA-seq
Marta Kovatcheva	Nature Communication (2017), Vol. 8	Liposarcoma LS8817 cells with doxorubicin vs quiescent(n=3/group)	Therapy induced senescence	-1.3684	6.2E-5	8.8617	12.5606	0.0003	Illumina HiSeq 2500, RNA-seq

Supplemental figure 1

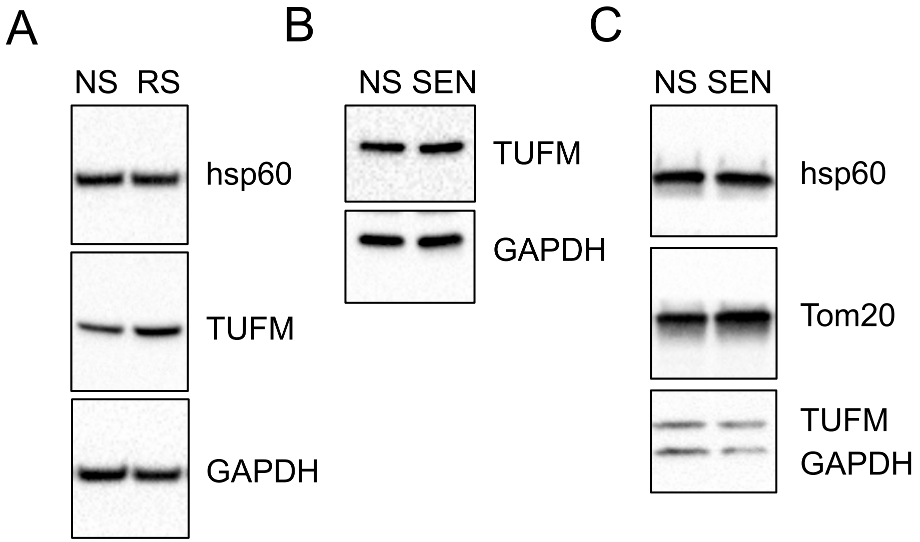

Supplemental figure 1. Mitochondrial protein expression in (A) replicative, (B) hydrogen peroxide-induced, and (C) doxorubicin-induced senescent HDFa cells. (n=3-4) Abbreviations: NS for Non-senescent cells (quiescent); RS for replicative-senescent cells; SEN for senescent cells.

Supplemental figure 2

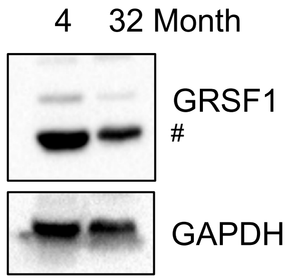

Supplemental figure 2. Additional western blots of GRSF1 in mouse skeletal muscle.

Supplement Figure 3

A. Figure 2C

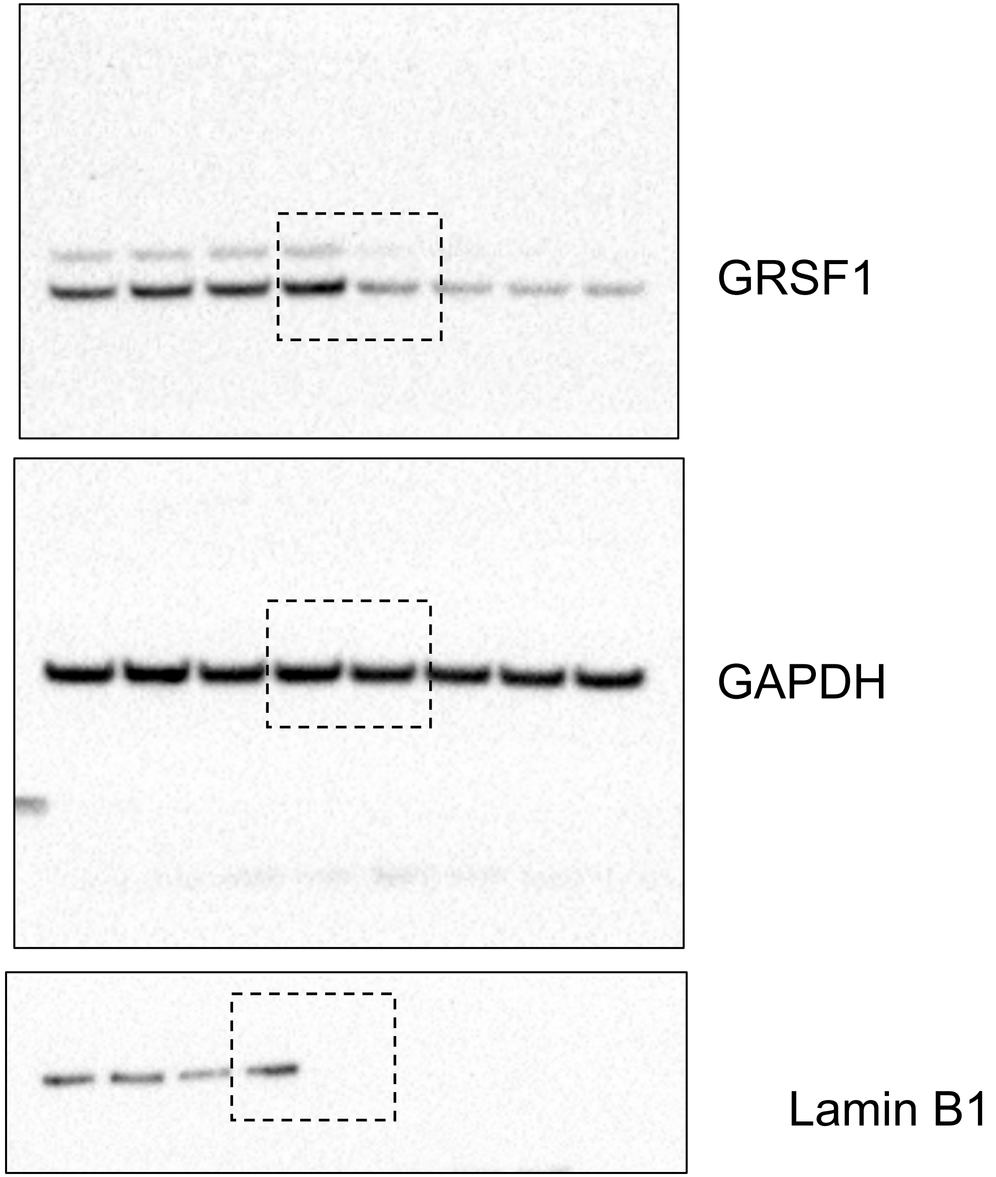

B. Figure 2G

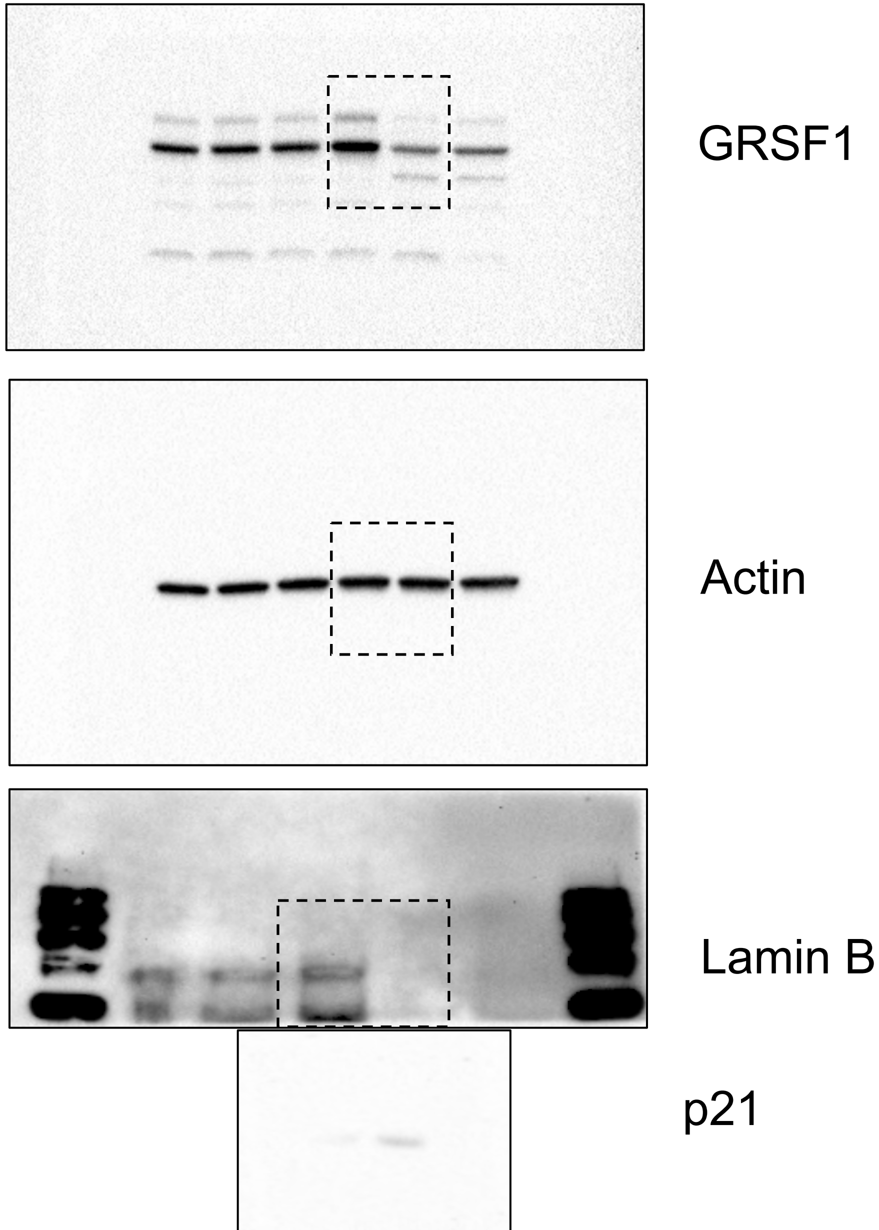

Supplement Figure 3

C. Figure 2K

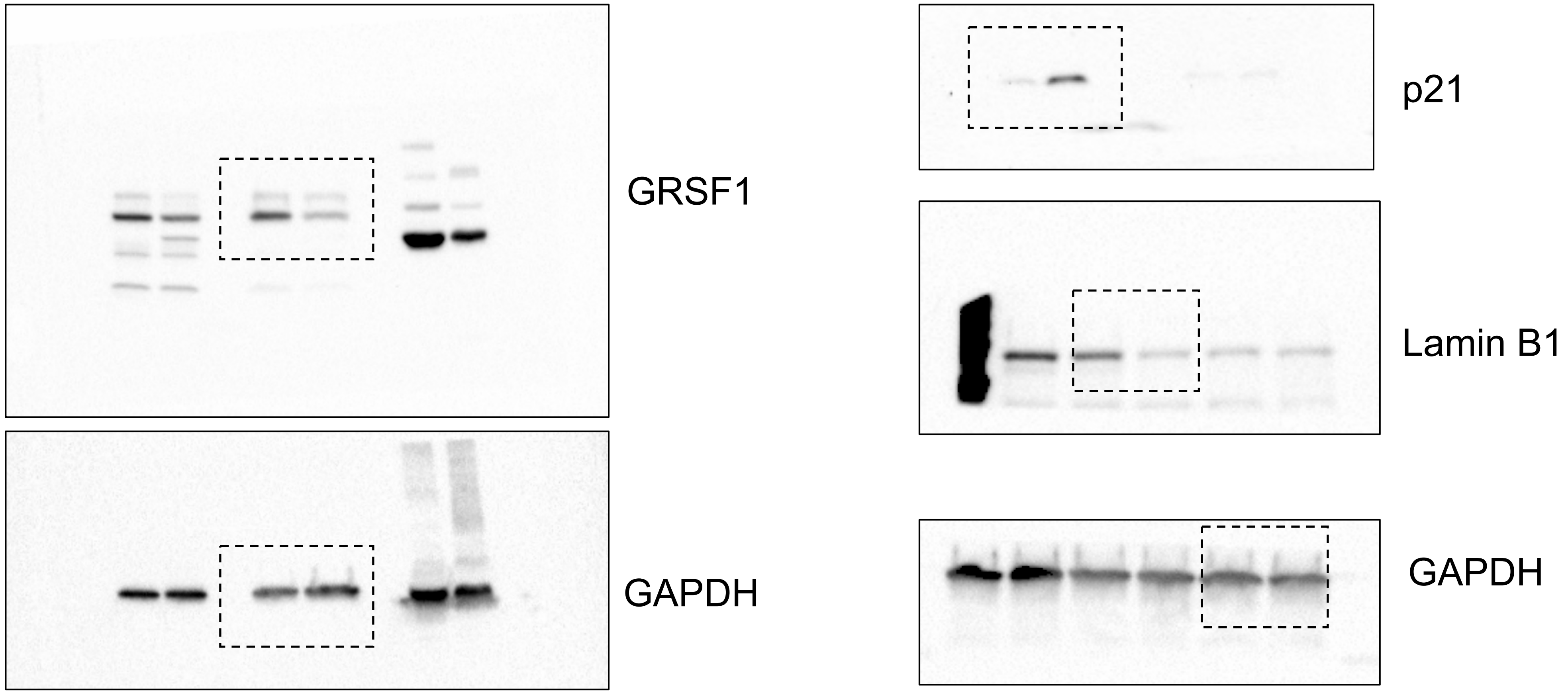

Supplement Figure 4

Figure 2M

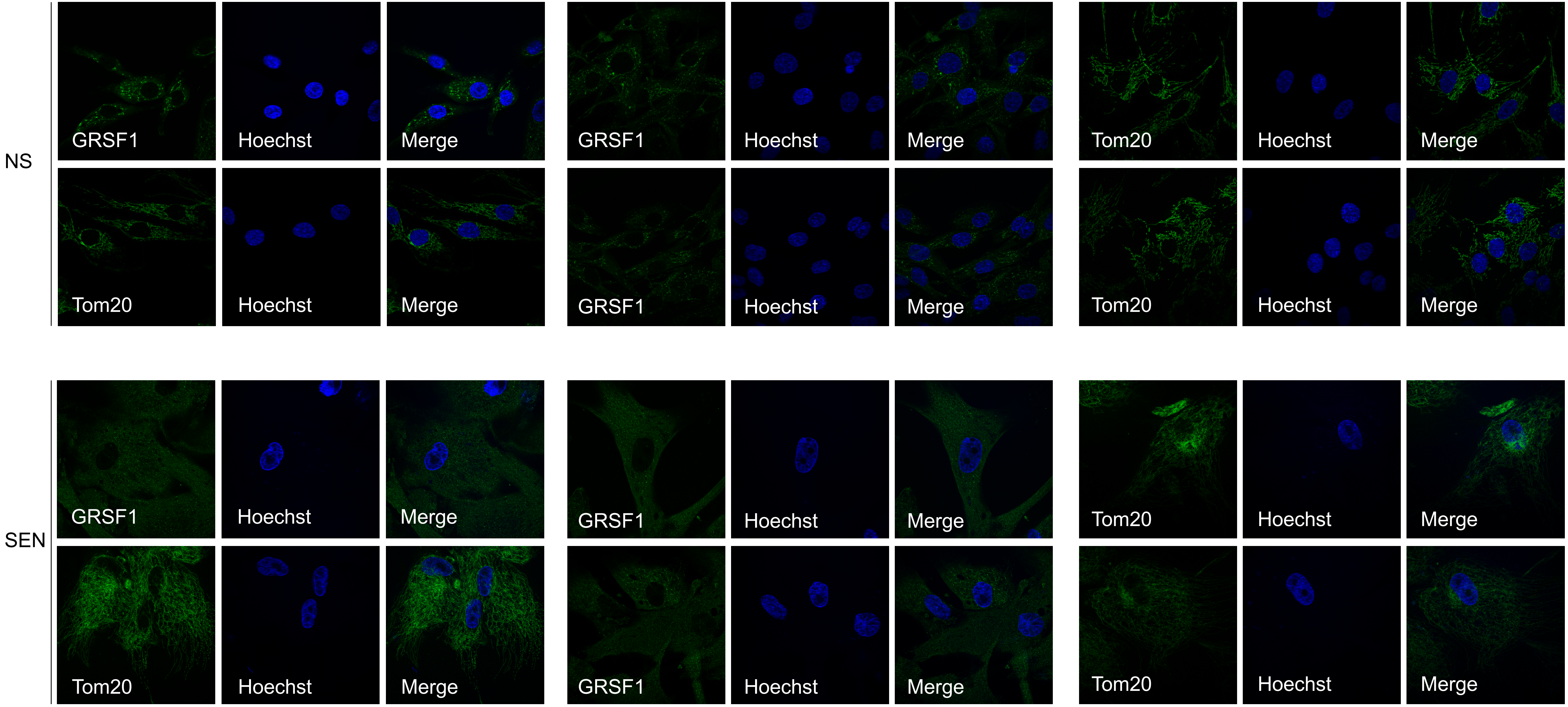

Supplement Figure 5

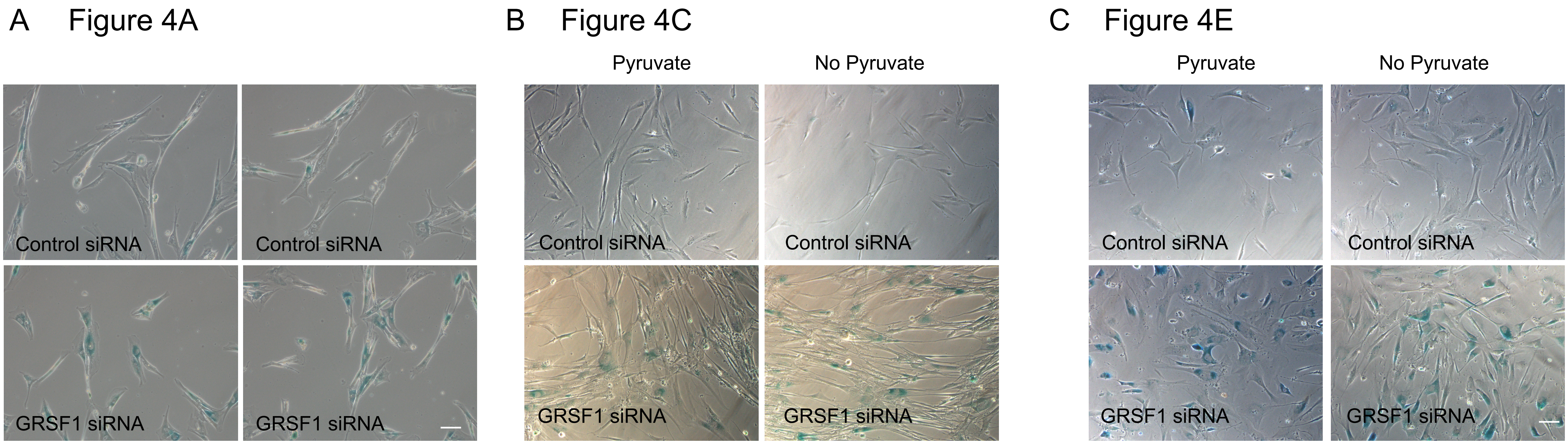

Supplement Figure 6

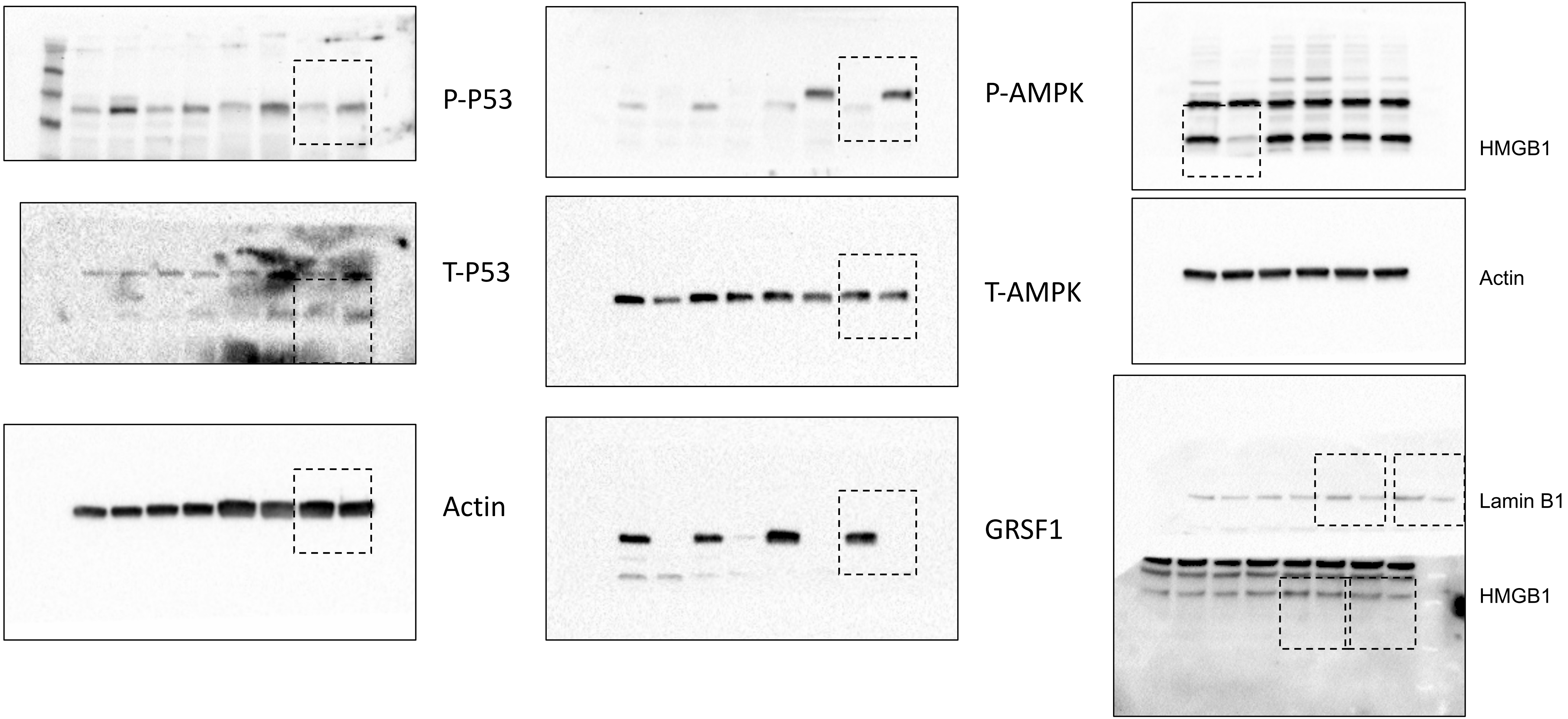

Supplement Figure 7

A Figure 4D

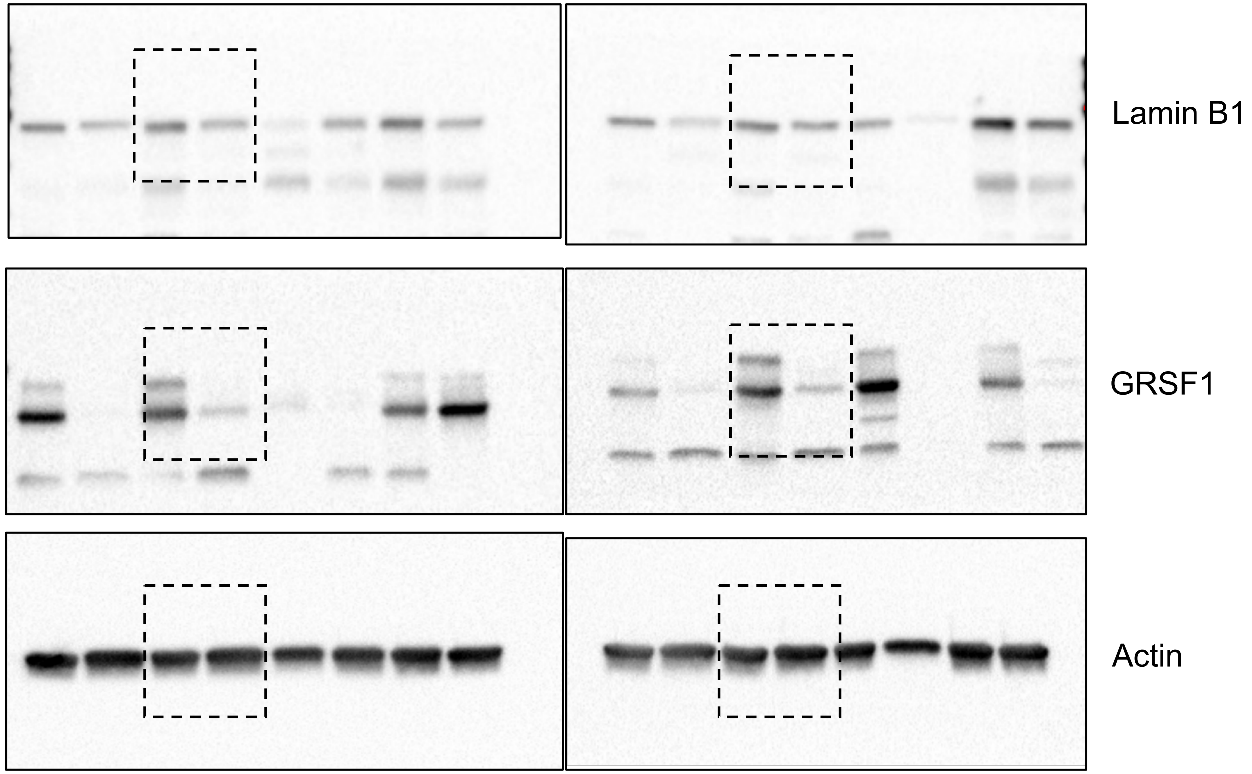

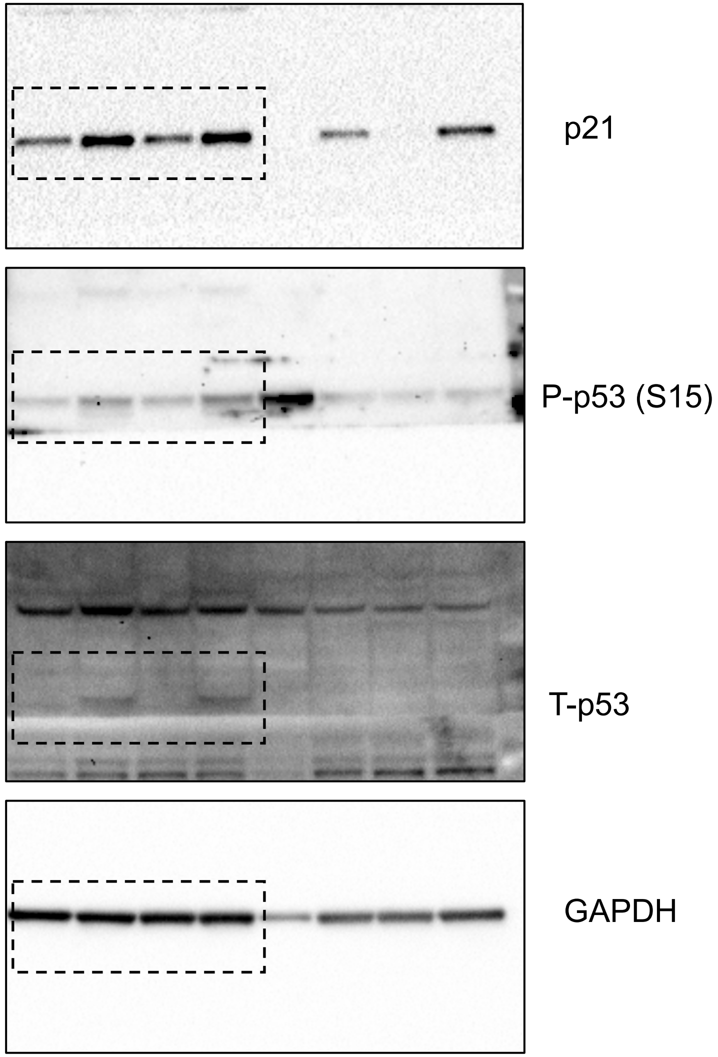

Supplement Figure 7

B Figure 4F

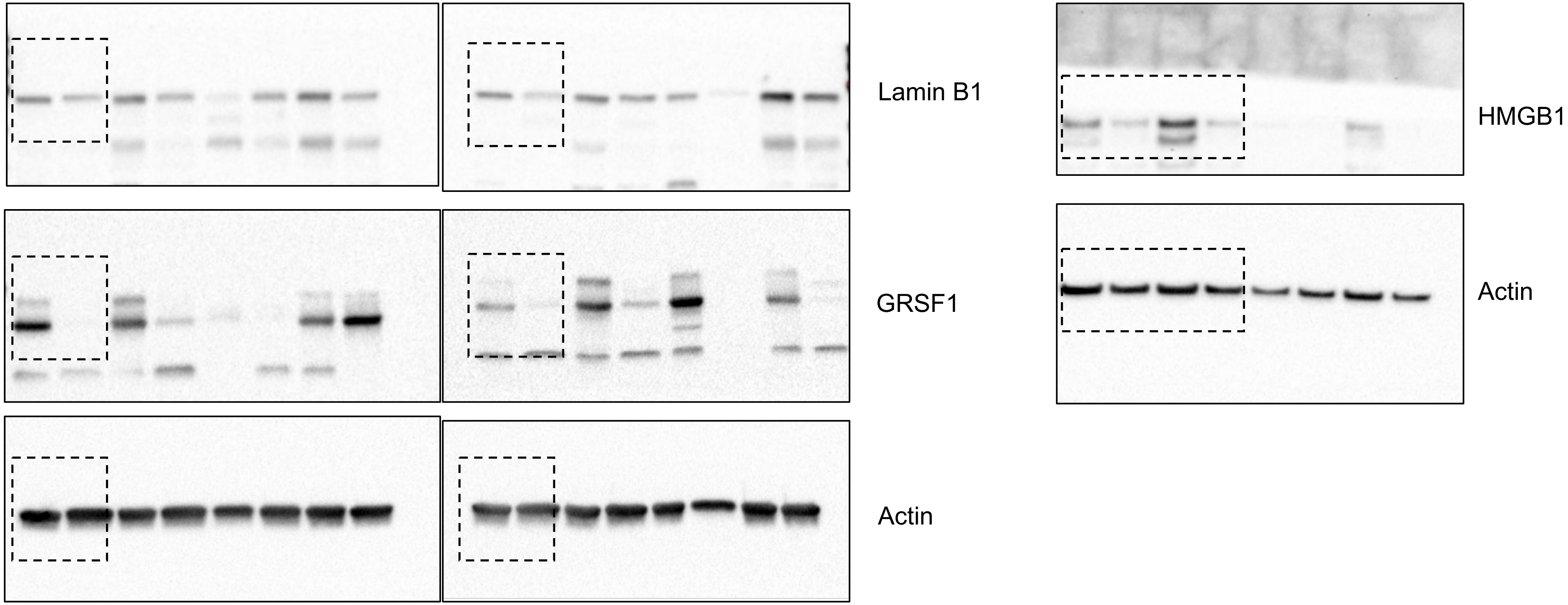

Supplement Figure 8

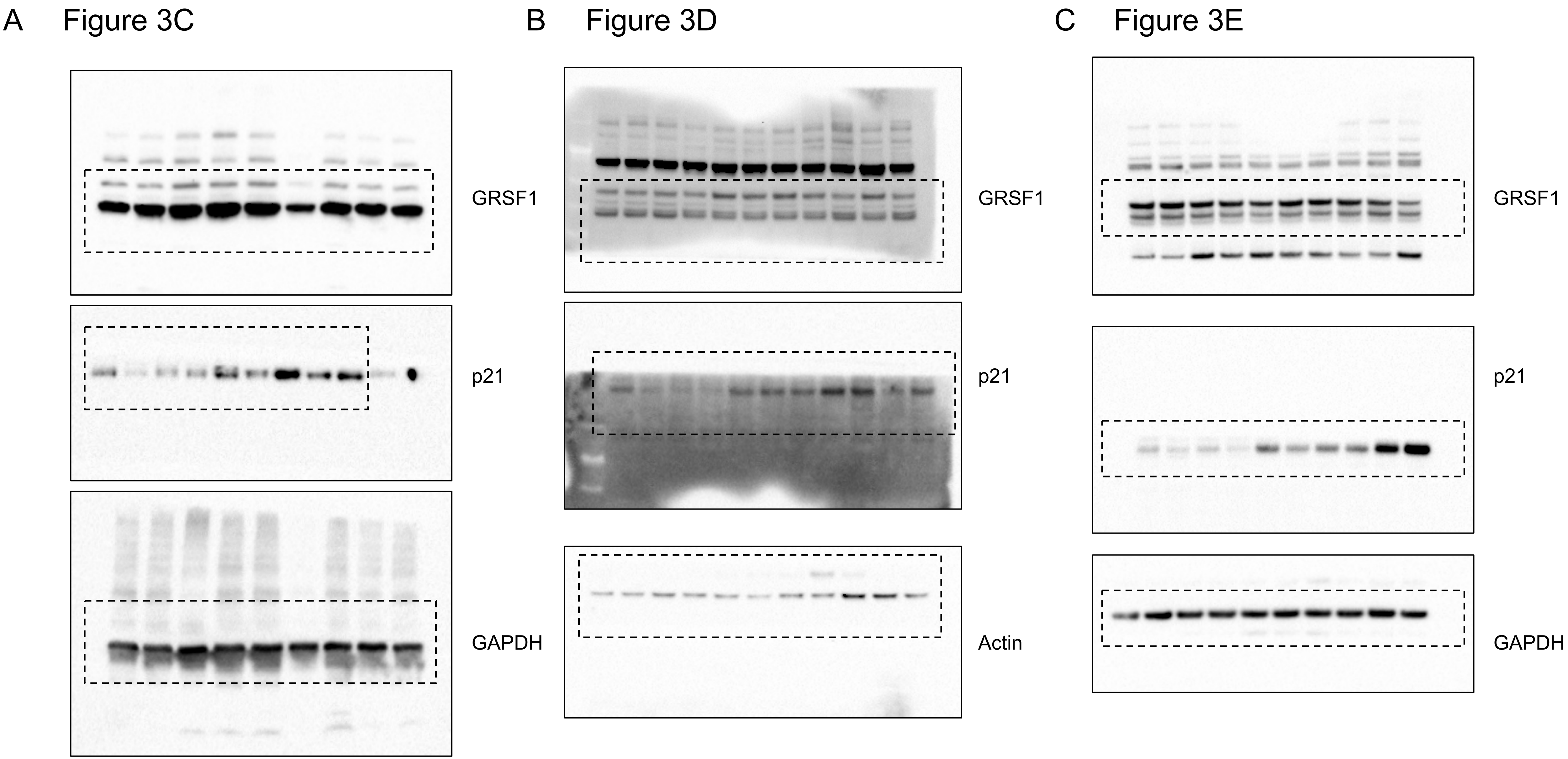

Supplement Figure 9

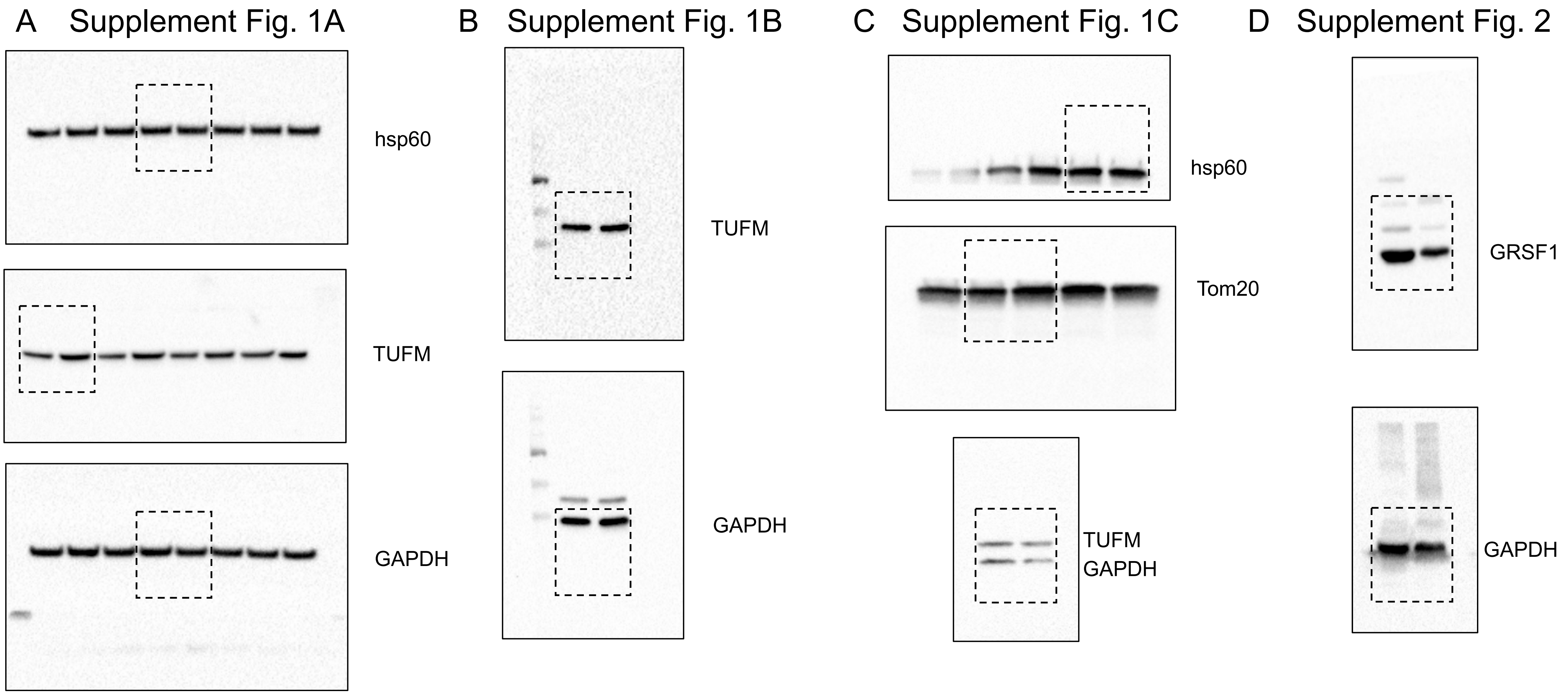

Supplement: Supplementary file 1 — Supplementary table and figures [file 41598_2019_42064_MOESM1_ESM.pdf]
